# Genetic polymorphisms in HLA-DP and STAT4 are associated with IgA nephropathy in a Southwest Chinese population

**DOI:** 10.18632/oncotarget.23829

**Published:** 2018-01-02

**Authors:** Bin Yang, Junlong Zhang, Xinle Liu, Zhuochun Huang, Zhenzhen Su, Yun Liao, Lanlan Wang

**Affiliations:** ^1^ Department of Lab Medicine, West China Hospital, Sichuan University, Sichuan 610041, Chengdu, China

**Keywords:** genetic polymorphisms, HLA-DP/DQ, STAT4, IgA nephropathy

## Abstract

IgA nephropathy (IgAN) is the most common chronic glomerular disease worldwide. Genetic factors are thought to be crucial in the pathogenesis of IgAN. However, few data are available on the relationship between human leucocyte antigen (HLA) and signal transducer and activator of transcription 4 (STAT4) polymorphisms and IgAN susceptibility in the Chinese population. Therefore, we examined HLA-DP/DQ and STAT4 polymorphisms (rs3077, rs9277535, rs7453920 and rs7574865) in a total of 630 subjects including 140 IgAN and 490 healthy controls in Chinese. There were significant associations between IgAN patients and healthy controls in the allele frequency of rs3077, rs9277535 and rs7574865. In addition, the genotypes of rs3077, rs9277535 and rs7574865 were also significantly associated with IgAN under recessive models. Moreover, the haplotypes block AAG, AGG, GAG and GGA in the HLA gene significantly correlated with the risk of IgAN. This is the first study demonstrating the significant associations of SNP rs3077, rs9277535 and rs7574865 and the haplotypes in the HLA gene with the risk of IgAN in a Southwest Chinese population. This research provides a new insight into the significant relationship between HLA-DP and STAT4 polymorphisms and the susceptibility to IgAN.

## INTRODUCTION

Immunoglobulin A nephropathy (IgAN) is the most common primary glomerulonephritis worldwide [1]. It is characterized by the deposition of IgA in glomerular mesangium and is diagnosed by immunohistochemical analysis on renal biopsies apart from other varying clinical manifestations [2]. The prevalence of IgAN is higher in Asia than in African ancestry and in Europeans [3, 4]. About 15% to 40% of patients will progress to end-stage renal failure (ESRF) requiring dialysis and/or kidney transplantation within 20 years of disease onset [5].

The precise etiology of IgAN is unclear, but genetic and environmental factors are thought to play important roles in the disease [6, 7]. Currently, increasing evidence support the importance of genetic factors, including familial clustering, differences in ethnic and geographical distributions, and variation of the clinical manifestations and prognosis [8–10]. Previous linkage studies have identified significant or suggestive loci on chromosomes 6q22–23, 4q26–31, 17q12–22, 2q36 and 3p24–23 [11–13], but the underlying genes remain elusive. Besides, the genome-wide association studies (GWASs) and candidate gene association studies have also revealed the susceptibility genes that contribute to IgAN, which include MHC, CFHR1, CFHR3 and DEFA [14, 15]. It is noteworthy that human leucocyte antigen (HLA) region contains the strongest common susceptibility alleles that predispose to IgA nephropathy in the European population [16]. Li, M. and Zhou, X. J., *et al.* have identified that the gene polymorphisms of HLA-DPB1, HLA-DRB1 and HLA-DRA were associated with the susceptibility to IgAN in a Chinese population [17–19]. In addition, HLA-DQ region gene polymorphism are reported to be associated with IgA nephropathy in in British Caucasoids [20, 21]. However, little has ever reported the possible connection between the HLA-DP/DQ gene polymorphisms and IgAN in a Chinese population. Thus, we investigate the correlation of the SNP rs3077 in HLA-DPA1, rs9277535 in HLA-DPB1 and rs7453920 in HLA-DQB2 with the susceptibility to IgAN in this study.

Furthermore, recent studies have strongly support the notion of shared genetics between immune-related diseases [19, 22]. And well established co-occurrences of systemic lupus erythematosus (SLE) with IgAN suggest common etiologic factors [23, 24]. Results of many genetic studies have identified the SNP rs7574865 in signal transducers and activators of transcription 4 (STAT4) gene was associated with an increased risk for diverse complex autoimmune diseases in different ethnic populations, such as SLE, rheumatoid arthritis and systemic sclerosis [25–27]. To our knowledge, there has been only one research that investigated the association of STAT4 gene polymorphisms with childhood IgAN [28]. However, little is known about the relationship between STAT4 gene polymorphisms and adult IgAN.

In the present study, we further analyze the associations of the four SNPs in HLA and STAT4 gene with the risk of IgAN. A comparative analysis between genotype distributions and pathological grade in the IgAN patients was also performed. Furthermore, we explore the linkage disequilibrium (LD) in HLA gene and the association between the HLA haplotypes and the susceptibility to IgAN in Southwest Chinese Han population.

## RESULTS

### Demographic and clinical characteristics

Demographic and clinical characteristics of the study subjects are described in Table 1. All of the study participants were Chinese. The patients were diagnosed as IgAN with renal biopsy at the Nephrology Department of West China Hospital and data was collected in detail. The mean age in IgAN patients and controls was 34 years and 38 years old respectively. Among all the IgAN patients, the median eGFR is 102.02 mL/min/1.73 m^2^ and the median proteinuria is 1.27 g/day. According to the Lee's glomerular grading system, the percent of Grade I and Grade II IgAN patients is 15.00% and 20.71% respectively (Table 1).

**Table 1:** Demographic and clinical characteristics of the study participants

Variables	Healthy controls	IgA nephropathy	*p*
*N*	490	140	
Age (years) ^†^	37.82 ± 9.89	33.79 ± 8.96	0.001^*^
Sex (male/female)	307/183	70/70	0.01^**^
BMI (kg/m^2^)^†^	-	23.35 ± 4.67	
SBP (mmHg) ^†^	-	121.25 ± 14.37	
DBP (mmHg) ^†^	-	75.9 ± 12.34	
Hemoglobin (g/L)^‡^	137 (132–143)	134 (118.75–148.25)	0.503^*^
Albumin (g/L)^‡^	48.8 (47.2–50.3)	40.29 (34.74–44.7)	0.001^*^
Creatinine (umol/L)^‡^	85.5 (74–95.75)	87.5 (72.25–112.9)	0.001^*^
BUN (mmol/L)^‡^	4.75 (4.19–5.56)	5.66 (4.38–6.99)	0.001^*^
eGFR (mL/min/1.73 m^2^)^‡^	-	102.02 (84.39–145.08)	
IgA (g/L)^‡^	-	2.60 (1.86–3.28)	
Serum Complement C3 (g/L)^‡^	-	0.92 (0.73–1.07)	
Serum Complement C4 (g/L)^‡^	-	0.21 (0.17–0.25)	
Proteinuria (g/day)^‡^	-	1.27 (0.54–2.73)	
Lee's glomerular grading system			
Grade I	-	21 (15.00%)	
Grade II	-	29 (20.71%)	
Grade III	-	53 (37.86%)	
Grade IV–V	-	37 (26.43%)	
The Oxford classification of tubular atrophy/interstitial fibrosis			
0–25%	-	126 (90.00%)	
26–50%	-	14 (10.00%)	

BMI: Body Mass Index; SBP: systolic blood pressure; DBP: diastolic blood pressure; BUN: blood urea nitrogen; eGFR: estimated glomerular filtration rate; IgA: immunoglobulin A; C3: complement 3; C4: complement 4.

^†^Data with normal distribution expressed as arithmetic mean ± standard deviation (SD);

^‡^Data with skew distribution were expressed as median (interquartile range);

^*^Statistical differences between groups tested using unpaired Student's *t* test;

^**^Statistical differences between groups tested using chi-square test.

### Association of HLA-DP/DQ and STAT4 Polymorphisms with the risk of IgAN

We genotyped four SNPs (rs3077 G/A, rs9277535 G/A, rs7453920 G/A and rs7574865 G/T) in 140 IgAN patients and 490 healthy controls (Supplementary Figure 1). The distributions of the allele frequencies for the four SNPs in the controls complied with Hardy-Weinberg equilibrium among Chinese Han population (*p* > 0.05). We analyzed the *p* values by adjusting age and gender to minimize their adverse effects. The results showed that there were significant associations between IgAN patients and healthy controls in the allele frequency of rs3077, rs9277535 and rs7574865 (*p* < 0.05). However, no significant difference was observed between IgAN patients and healthy controls in the allele frequency of HLA-DQB2 rs7453920 (Table 2).

**Table 2:** Distribution of HLA-DP/DQ and STAT4 alleles in IgA nephropathy patients and healthy controls

Genes	SNPs	Alleles	Controls (%)	IgAN (%)	Crude OR (95% CI)	*P*	Adjusted OR (95% CI)	p^*^
HLA-DP	rs3077	Allele G	658 (67.1)	157 (56.1)	1.00 (ref.)		1.00 (ref.)	
		Allele A	322 (32.9)	123 (43.9)	**1.60 (1.22–2.09)**	**0.001**	**1.69 (1.28–2.24)**	**2.2E-04**
		HWE	*p* = 0.93	*p* =1.9E-15				
HLA-DP	rs9277535	Allele G	610 (62.2)	128 (45.7)	1.00 (ref.)		1.00 (ref.)	
		Allele A	370 (37.8)	152 (54.3)	**1.96 (1.49–2.56)**	**1.0E-06**	**1.99 (1.52–2.63)**	**1.0E-06**
		HWE	*p* = 0.99	*p* = 0.01				
HLA-DQ	rs7453920	Allele G	855 (87.2)	251 (89.6)	1.00 (ref.)		1.00 (ref.)	
		Allele A	125 (12.8)	29 (10.4)	0.79 (0.51–1.21)	0.28	0.70 (0.45–1.08)	0.11
		HWE	*p* = 0.26	*p* = 0.39				
STAT4	rs7574865	Allele G	652 (66.5)	160 (57.1)	1.00 (ref.)		1.00 (ref.)	
		Allele T	328 (33.5)	120 (42.9)	**1.49 (1.14–1.96)**	**0.004**	**1.60 (1.21–2.12)**	**0.001**
		HWE	*p* =0.82	*p* = 1.9E-08				

OR: odds ratio; CI: confidence interval; HWE: Hardy-Weinberg equilibrium.

^*^Logistic regression controlling for age and sex.

Bold values are statistically significant (*p* < 0.05).

Besides, we tested for association under different inheritance models by using logistic regression adjusted for age and gender (Table 3). We found that the genotypes of rs3077, rs9277535 and rs7574865 were significantly associated with IgAN under recessive models (rs3077, adjusted OR = 5.63, 95 % CI = 3.48–9.10, adjusted *p* = 1.00E-06; rs9277535, adjusted OR = 2.97, 95% CI = 1.89–4.67, adjusted *p* = 3.00E-06; rs7574865, adjusted OR =3.79, 95 % CI = 2.36–6.10, adjusted *p* = 1.00E-06). In addition, the genotype of rs9277535 was also significantly associated with IgAN under dominant model (adjusted OR = 1.28, 95% CI = 1.10–1.48, adjusted *p* = 0.001) (Table 3).

**Table 3:** Association of HLA-DP/DQ and STAT4 polymorphisms in IgA nephropathy patients and healthy controls

SNPs	Model	genotypes	Controls (%)	IgAN (%)	Crude OR (95% CI)	*P*	Adjusted OR (95% CI)	p^*^
rs3077	Codominant	GG	219 (44.7)	68 (48.6)	1.00 (ref.)		1.00 (ref.)	
		AG	220 (44.9)	21 (15.0)	**0.31 (0.18–0.52)**	**5.0E-06**	**0.32 (0.19–0.54)**	**2.7E-05**
		AA	51 (10.4)	51 (36.4)	**3.22 (2.01–5.17)**	**1.0E-06**	**1.91 (1.49–2.45)**	**1.0E-06**
	Dominant	GG	219 (44.7)	68 (48.6)	1.00 (ref.)		1.00 (ref.)	
		AG + AA	271 (55.3)	72 (51.4)	0.85 (0.58–1.24)	0.406	0.97 (0.85–1.10)	0.61
	Recessive	GG + AG	439 (89.6)	89 (63.6)	1.00 (ref.)		1.00 (ref.)	
		AA	51 (10.4)	51 (36.4)	**1.53 (1.07–2.18)**	**0.018**	**5.63 (3.48–9.10)**	**1.0E-06**
rs9277535	Codominant	GG	190 (38.8)	33 (23.6)	1.00 (ref.)		1.00 (ref.)	
		AG	230 (46.9)	62 (44.3)	**1.55 (0.97–2.47)**	**0.06**	**1.55 (0.97–2.50)**	**0.07**
		AA	70 (14.3)	45 (32.1)	**3.70 (2.19–6.26)**	**1.0E-06**	**1.97 (1.50–2.58)**	**1.0E-06**
	Dominant	GG	190 (38.8)	33 (23.6)	1.00 (ref.)		1.00 (ref.)	
		AG + AA	300 (61.2)	107 (76.4)	**2.05 (1.34–3.16)**	**0.001**	**1.28 (1.10–1.48)**	**0.001**
	Recessive	GG + AG	420 (85.7)	95 (67.9)	1.00 (ref.)		1.00 (ref.)	
		AA	70 (14.3)	45 (32.1)	**2.84 (1.84–4.39)**	**1.0E-06**	**2.97 (1.89–4.67)**	**3.0E-06**
rs7453920	Codominant	GG	377 (76.9)	114 (81.4)	1.00 (ref.)		1.00 (ref.)	
		AG	101 (20.6)	23 (16.4)	0.75 (0.46–1.24)	0.26	0.70 (0.42–1.18)	0.18
		AA	12 (2.4)	3 (2.1)	0.83 (0.23–2.98)	0.77	0.74 (0.38–1.44)	0.38
	Dominant	GG	377 (76.9)	114 (81.4)	1.00 (ref.)		1.00 (ref.)	
		AG + AA	113 (23.1)	26 (18.6)	0.76 (0.47–1.22)	0.26	0.88 (0.75–1.04)	0.13
	Recessive	GG + AG	478 (97.6)	137 (97.9)	1.00 (ref.)		1.00 (ref.)	
		AA	12 (2.4)	3 (2.1)	0.87 (0.24–3.14)	0.83	0.60 (0.16–2.22)	0.44
rs7574865	Codominant	GG	220 (44.9)	63 (45.0)	1.00 (ref.)		1.00 (ref.)	
		TG	212 (43.3)	34 (24.3)	**0.56 (0.35–0.88)**	**0.01**	**0.57 (0.36–0.91)**	**0.02**
		TT	58 (11.8)	43 (30.7)	**2.59 (1.59–4.20)**	**8.8E-06**	**1.76 (1.36–2.27)**	**1.8E-05**
	Dominant	GG	220 (44.9)	63 (45.0)	1.00 (ref.)		1.00 (ref.)	
		TG + TT	270 (55.1)	77 (55.0)	1.00 (0.68–1.45)	0.98	1.02 (0.89–1.16)	0.77
	Recessive	GG + TG	432 (88.2)	97 (69.3)	1.00 (ref.)		1.00 (ref.)	
		TT	58 (11.8)	43 (30.7)	**3.30 (2.10–5.19)**	**1.0E-06**	**3.79 (2.36–6.10)**	**1.0E-06**

OR: odds ratio; CI: confidence interval.

^*^Logistic regression controlling for age and sex.

Bold values are statistically significant (*p* < 0.05).

### Stratification analyses of HLA-DP/DQ and STAT4 polymorphisms and risk for IgAN

Based on the Lee's glomerular grading system, we divided all the IgAN patients into two groups: Lee's Grade I-II and Lee's Grade III-V. Similar genotype distributions of HLA-DP/DQ and STAT4 polymorphisms were found between the two groups (dominant model: rs3077, OR = 0.85, 95% CI = 0.43–1.70, *p* = 0.65; rs9277535, OR = 0.49, 95% CI = 0.20–1.20, *p* = 0.12; rs7453920, OR = 0.86, 95% CI =0.36–2.08, *p* = 0.75; rs7574865, OR = 0.94, 95% CI = 0.47–1.88, *p* = 0.86) (Table 4).

**Table 4:** Association of HLA-DP/DQ and STAT4 polymorphisms with clinical characteristics in patients with IgA nephropathy

SNPs	Lee's Grade I–II (%)	Lee's Grade III–V (%)	OR (95% CI)	*p*
rs3077				
GG	23 (46.0)	45 (50.0)	1.00 (ref.)	
AG + AA	27 (54.0)	45 (50.0)	0.85 (0.43–1.70)	0.65
rs9277535				
GG	8 (16.0)	25 (27.8)	1.00 (ref.)	
AG + AA	42 (84.0)	65 (72.2)	0.49 (0.20–1.20)	0.12
rs7453920				
GG	40 (80.0)	74 (82.2)	1.00 (ref.)	
AG + AA	10 (20.0)	16 (17.8)	0.86 (0.36–2.08)	0.75
rs7574865				
GG	22 (44.0)	41 (45.6)	1.00 (ref.)	
TG + TT	28 (56.0)	49 (54.4)	0.94 (0.47–1.88)	0.86

OR: odds ratio; CI: confidence interval.

### Haplotype analysis of HLA-DP/DQ with the risk of IgAN

**Figure 1:**
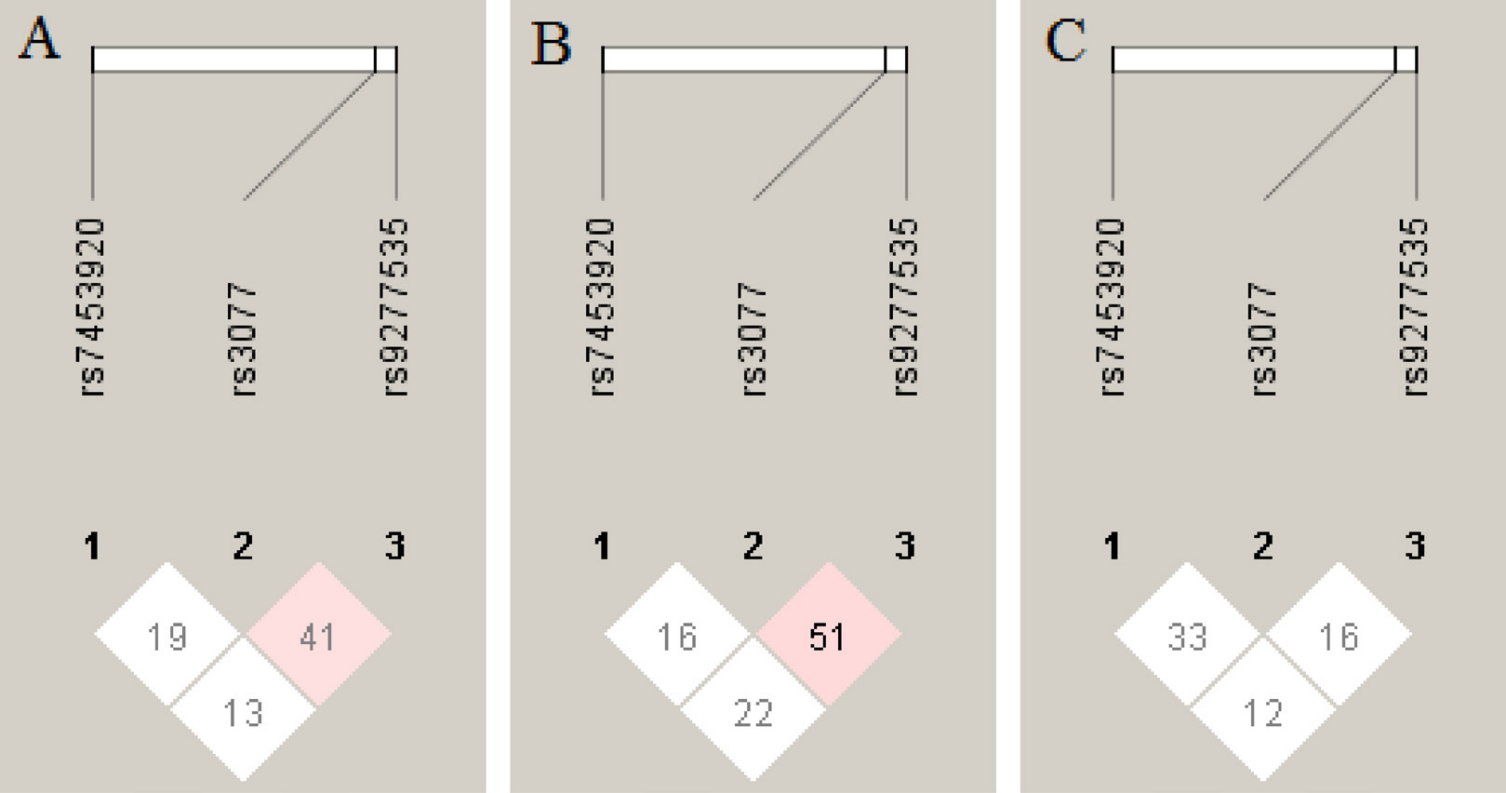
Linkage disequilibrium (LD) analysis of the HLA-DPA1, HLA-DPB1 and HLA-DQB2 SNPs (**A**) LD analysis of HLA gene in all the subjects. (**B**) LD analysis of HLA gene in healthy controls. (**C**) LD analysis of HLA gene in IgA nephropathy patients. The LD status is expounded by the D’ value.

Haplotypes were constructed on the basis of three SNPs (rs3077, rs9277535 and rs7453920). Both HLA haplotype blocks in IgAN patients and healthy controls were constructed from rs3077 (minor allele A), rs9277535 (minor allele A) and rs7453920 (minor allele A) (Figure 1). There was no strong linkage disequilibrium among the three SNPs (rs7453920, rs3077 and rs9277535). D′ < 0.6 and *r*^2^ < 0.3 in all the blocks (Figure 1). We found that haplotype block AAG, AGG, GAG and GGA significantly correlated with reduced risk of IgAN (AAG: OR = 1.42, 95% CI = 1.03–1.96, *p* = 0.03; AGG: OR = 1.59, 95% CI =1.07–2.36, *p* = 0.02; GAG: OR = 2.34, 95% CI =1.69–3.24, *p* = 1.67E-07; GGA: OR = 0.46, 95% CI = 0.22–0.99, *p* = 0.04), while haplotype block AAA showed no significance with the risk of IgAN (Table 5).

**Table 5:** Haplotype analysis for genotypes of HLA-DP/DQ and IgAN risk

Haplotypes	Case (freq.)	Control (freq.)	OR (95%CI)	*p*
AAA	11.50 (0.041)	50.15 (0.051)	0.81 (0.42~1.56)	0.526019
AAG	64.41 (0.230)	174.03 (0.178)	**1.42 (1.03~1.96)**	**0.033938**
AGG	40.29 (0.144)	95.49 (0.097)	**1.59 (1.07~2.36)**	**0.020488**
GAG	73.31 (0.262)	131.98 (0.135)	**2.34 (1.69~3.24**)	**1.67E-07**
GGA	7.91 (0.028)	58.67 (0.060)	**0.46 (0.22~0.99)**	**0.041992**

OR: odds ratio; CI: confidence interval; IgAN: IgA nephropathy.

Order of HLA haplotype block: rs3077 (minor allele A), rs9277535 (minor allele A) and rs7453920 (minor allele A).

Bold values are statistically significant (*p* < 0.05).

## DISCUSSION

IgAN is regarded as a complex disease that is initiated by more than one genetic factor combined with environmental factors [29]. Over the past few years, genome-wide association studies (GWASs) have successfully identified numerous genetic variants for complex human diseases [14–16]. Although the pathogenesis of IgAN is ambiguous, the associations between genes polymorphisms and IgAN susceptibility increase our understanding of the mechanisms. In this study, we noted that rs3077 G/A, rs9277535 G/A and rs7574865 G/T polymorphisms were significantly associated with the risk of IgAN. However, no significant association was observed between HLA-DQ rs7453920 polymorphism and IgAN susceptibility. In addition, we found that HLA haplotype blocks AAG, AGG and GAG are significantly correlated with increased risk of IgAN. To the best of our knowledge, this is the first report demonstrating the significant associations of rs3077 G/A, rs9277535 G/A, rs7574865 G/T polymorphisms and haplotypes with IgAN in Southwest Chinese population.

Achievements have been made in the last decade by investigations, supporting the involvement of HLA class II molecules in the development of IgAN [17–20]. Li, M., *et al.* identified that the novel HLA-DPB1^*^1702 allele was associated with IgAN patient of Han ethnic in China [17]. Zhou, X. J., *et al.* found that HLA-DRA rs9501626 and HLA-DRB1 rs9271366 were significantly associated with IgAN [19]. In this study, individuals with minor alleles of HLA-DP rs3077 and rs9277535 had significantly higher risk of developing IgAN, mostly with an recessive genetic effect, suggesting that the minor alleles of these SNPs in HLA-DP gene might be a risk genetic factor of IgAN. Considering that the differences were present for associated genes not having linkage disequilibrium, the SNPs were independently contributed to the risk of IgAN.

Above results again suggested that HLA was involved in the pathogenesis of IgAN. Following are some possible mechanisms. Firstly, some HLA alleles may have a permissive role in autoimmunity. Secondly, MHC class II molecules may participate in the regulation of intestinal inflammation and IgA production [30]. Although the exact mechanism is unclear, these genotyping data identifies a new genetic risk factor for IgAN. The prevalence of IgAN varies greatly among different ethnicities, being higher in Asians but lower in Africans [31]. Further systematic resequencing of this genomic region in various races and *in vitro* functional genomic studies were warranted to reveal the exact mechanism.

STAT4 is one of STAT family proteins, and plays a central role in the related cytokine signaling [32, 33]. Accumulated evidence have suggested that STAT4 are involved in the pathogenesis of renal disorders [34, 35]. Of note, SLE patients having clinical signs of nephritis show a strong association with STAT4 [36]. Hahn, W. H., *et al.* also suggested that polymorphisms of STAT1 and STAT4 are associated with increased susceptibility, pathological advancement, and development of proteinuria in childhood IgAN [28]. Interestingly, we also found that there are significant differences in allele frequencies and genotype distribution between IgAN patients and healthy controls in STAT4 rs7574865. The results in our study are similar with the previous studies.

As we all know, IgAN is mainly due to an abnormal immune response, characterized by increased synthesis of deglycosylated IgA1. In addition, T-helper lymphocytes play an important role in the mucosal effect in IgAN [37]. Considering the important role of STAT4 polymorphisms in other immune-related disease, we speculated that STAT4 contribute to the risk of IgA with the possible reason such as triggering antigens exposure and inadequately enhanced immune response. The common gene polymorphisms identified in IgAN and other immunology disease, could improve the understanding of pathogenesis and therapy.

In addition, previous studies have demonstrated that clinical characteristics of IgAN patients were also significantly associated with gene polymorphisms. Hahn, W.H., *et al.* found that SNP frequencies were significantly different between patients with pathologically mild and advanced disease in STAT1 rs6718902 and STAT4 rs7561832 [28]. Therefore, we performed stratification analyses of HLA-DP/DQ and STAT4 polymorphisms and risk for IgAN. However, no associations between the severity of IgAN and these genotypes were found in this study. Considering the small sample size of the IgAN subgroups in our study, more attention is needed to be paid to the stratification analysis in future.

There were several limitations in our study. Firstly, there is a selection bias due to the enrolment of study subjects based on the hospital. Secondly, the number of IgAN patients is limited; hence the subgroup analysis of different disease severity is restricted. Thirdly, there are different environmental factors between the Chinese population and other ethnic populations, especially the gene-environment interactions. Finally, the present study assessed only the associations between gene polymorphisms and IgA nephropathy; biologically plausible mechanisms such as functional genomic studies have not been analyzed, which made it difficult to discern their exact contribution to IgAN proneness.

In summary, this is the first study that demonstrated the significant associations of HLA-DPA1 rs3077, HLA-DPB1 rs9277535, STAT4 rs7574865 with IgAN susceptibility in a Chinese Han population. Moreover, this report also revealed that LD blocks around HLA-DP/DQ are strongly associated with the risk of IgAN. The significant relationship between the SNPs in the HLA-DP and STAT4 gene and the risk of IgAN broaden our understanding of the importance of the genetic role in IgAN. Further analysis in different ethnic populations as well as functional experiments will be needed to validate the results.

## MATERIALS AND METHODS

### Subjects

The study group consisted of 630 subjects, including 140 IgAN patients and 490 healthy controls. All the subjects were recruited from West China Hospital of Sichuan University. The 140 IgAN patients were diagnosed with biopsy-proven, defined by standard criteria, including predominant, or codominant mesangial deposition of IgA, regardless of treatment given. Exclusion criteria were hepatic disease, diabetes mellitus, parathyroid disease, hypertension or other inflammatory disease. Members of the control group, who had not any personal or family history of either renal diseases or other diseases, were chosen from the Health Examination Center of West China Hospital. The study was approved by the Institutional Ethics Committee of West China Hospital of Sichuan University and complied with the Declaration of Helsinki. Written informed consent was obtained prior to enrollment from all subjects.

### Measurement of clinical parameter

Blood samples were collected at the fasting state from each subject. IgAN patients were proven by renal biopsy, demonstrating a dominant IgA deposition in the mesangium by immunofluorescence microscopy. Clinical characteristics of patients with IgAN were collected at the same time of kidney biopsy, including age of onset, the course of disease, clinical symptoms, body mass index (BMI), systolic/diastolic blood pressure, hemoglobin, serum albumin, serum creatinine, blood urea nitrogen (BUN), 24-h urine protein, estimated glomerular filtration rate (eGFR), immunoglobulin A (IgA), Complement C3, C4, and Lee's pathological grade. And eGFR was analyzed by using a modification of the modified diet in renal disease equation based on the Chinese chronic kidney disease (CKD) [38]. Moreover, pathology grading was recorded for each individual.

### HLA-DP/DQ and STAT4 polymorphisms

Four milliliters of peripheral blood were collected from each subject. DNA was extracted from peripheral blood samples by using the Genomic DNA kit (Biotake Corporation, Beijing, China) and the concentration was measured by NanoDrop 2000 c spectrophometer (Thermo Scientific, DE, USA). The extracted DNA was detected immediately or stored at −80°C for less than 6 months.

The information of SNPs in HLA-DP/DQ and STAT4 genes were showed in Supplementary Table 1. Primers for the four SNPs were shown in Supplementary Table 2. All the four SNPs were genotyped using polymerase chain reaction high resolution melting (HRM) analysis performed on Light Cycler 480 (Roche Diagnostics, Penzberg, Bavaria, Germany) (Supplementary Figure 1). SNP genotyping was performed in a 10 μl reaction system contained 5 μl Roche Master Mix (Roche Applied Science, Mannheim, Germany) which comprises FastStart Taq DNA Polymerase and the High Resolution Melting Dye in a reaction buffer, 1.2 μl 25 mM MgCl_2_, 0.1 μl 10 μmol/L Forward Primer and 0.1 μl 10 μmol/L Reverse Primer, 2.6 μl deionized water and finally 1 μl DNA sample. The whole genotyping process encompasses four programmes, namely, predenaturation, amplification, high resolution melting and cooling. When finished, the results were analyzed by the corresponding Gene Scanning Software v1.2 (Roche Diagnostic, Germany). All the data were shown in Supplementary Dataset File 1.

### Statistical analysis

Hardy-Weinberg equilibrium (HWE) was independently tested for each polymorphism. Normally distributed variables in the clinical characteristics of the participants were expressed as mean ± SD and were compared between groups by independent *t* test. Continuous variables with skewed distribution were described with median and interquartile.

Gender distribution, genotype and allele proportions between patients and controls, and associations between the SNPs and clinical characteristics of IgAN patients were done by Pearson's chi-square test. The effect of SNPs was tested for odds ratio (OR), 95% confidence interval (CI), and *p*-values. To reduce the additive effect of age and gender, all the tests were adjusted for age and gender by binary logistic regression. Genetic models were defined relative to the minor allele. We introduced different genetic models (codominant, dominant and recessive models) to measure the associations between the genes and risk of IgAN. Haplotype analysis was also performed to explore whether HLA-DP/DQ polymorphisms were in strong linkage disequilibrium (LD) with other gene locus or they independently contributed to the risk of IgAN. Haplotypes with frequencies of more than 0.03 were analyzed. A linkage disequilibrium (LD) block of polymorphisms was tested using Haploview software [39]. A two-sided *p* value of less than 0.05 was deemed as statistically significant.

All the statistical analyses were performed using SPSS 21.0 software (IBM Corporation, New York, USA) and SHEsis online software (http://analysis.bio-x.cn/myAnalysis.php) [40]. Additional information on statistical methods is available in the Supplementary Material.

## SUPPLEMENTARY MATERIALS FIGURES AND TABLES




