# Supplementary material for: Genetic polymorphisms in HLA-DP and STAT4 are associated with IgA nephropathy in a Southwest Chinese population
Source: Oncotarget. 2018 Jan 2;9(6):7066–74. doi: 10.18632/oncotarget.23829 (PMC5805536; doi:10.18632/oncotarget.23829)
Supplement: Supplementary file 1 [file oncotarget-09-7066-s001.pdf]

Genetic polymorphisms in HLA-DP and STAT4 are associated with IgA nephropathy in a Southwest Chinese population

SUPPLEMENTARY MATERIALS

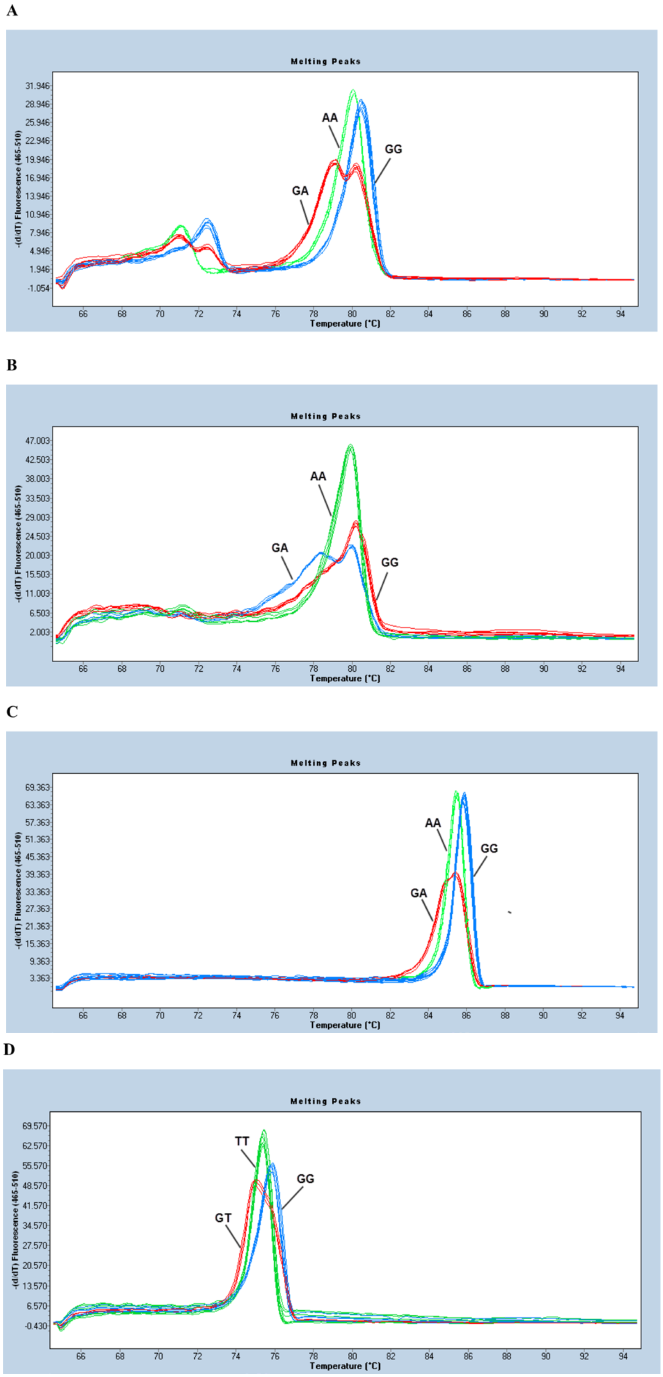

Supplementary Figure 1: The genotypes of the four SNPs in HLA-DP/DQ and STAT4 by high resolution melting (HRM). (**A**) HLA-DPA1 rs3077 G/A, (**B**) HLA-DPB1 rs9277535 G/A, (**C**) HLA-DQB2 rs7453920 G/A, (**D**) STAT4 rs7574865 G/T.

Supplementary Table 1: Information of SNPs in HLA-DP/DQ and STAT4 genes

Gene	SNP	Chr. Location	Region in gene	Alleles*	MAF
HLA-DPA1	rs3077	6:33141000	3′-UTR	G/A	0.383
HLA-DPB1	rs9277535	6:33162839	3′-UTR	G/A	0.478
HLA-DQB2	rs7453920	6:32837990	Intron	G/A	0.122
STAT4	rs7574865	2:191099907	Intron	G/T	0.349

Chr: chromosome; MAF: minor allele frequency from data of Han Chinese in the 1000 Genome Project. UTR: Untranslated Regions.
*Major allele/minor allele.

Supplementary Table 2: **Sequences of primers used for systematic search for SNPs in HLA-DP/DQ and STAT4**

SNP/rs number	primer	Sequence
HLA-DP rs3077	Forward	5′-TCAGCTTTTCTTCTCACTTCATGTG-3′
	Reverse	5′-GAGCTTGAAGGGTCAGCAATTC-3′
HLA-DP rs9277535	Forward	5′-AATGGTGAGCAGACTGCAAATCT-3′
	Reverse	5′-TGGTAATGATAAAACATGCTCTCAGTAA-3′
HLA-DQ rs7453920	Forward	5′-TTTAGGGAGGTAAGAGGGAAAGC-3′
	Reverse	5′-CGAGAACGCCCTGATCTAAGA-3′
STAT4 rs7574865	Forward	5′-AAAGAAGTGGGATAAAAAGAAGTTTG-3′
	Reverse	5′-CCACTGAAATAAGATAACCACTGT-3′

Supplementary Dataset File 1: All brief data. See Supplementary_Dataset File_1
